# Apixaban as a Rare Cause of Leukocytoclastic Vasculitis

**DOI:** 10.1155/2020/7234069

**Published:** 2020-02-26

**Authors:** Jenna Spears, David Alexandre Chetrit, Sina Manthey, Christopher Lee, Yousif Al-Saiegh

**Affiliations:** Department of Medicine, Pennsylvania Hospital, University of Pennsylvania Health System (UPHS), Philadelphia, PA, USA

## Abstract

Apixaban is a rare cause of leukocytoclastic vasculitis (LCV). To our knowledge, there is only one other reported case due to apixaban in the literature. We present a case of apixaban-induced leukocytoclastic vasculitis in a 95-year-old male. He had been started on apixaban 12 days prior to presentation and developed worsening palpable purpura of his lower extremities. Possible etiologies of this new rash were excluded, with biopsy showing extensive purpura with superficial perivascular neutrophilic infiltrate and leukocytoclasis. Apixaban was discontinued, and the patient was started on a slow prednisone taper with subsequent resolution of his rash.

## 1. Introduction

**Figure 1:**
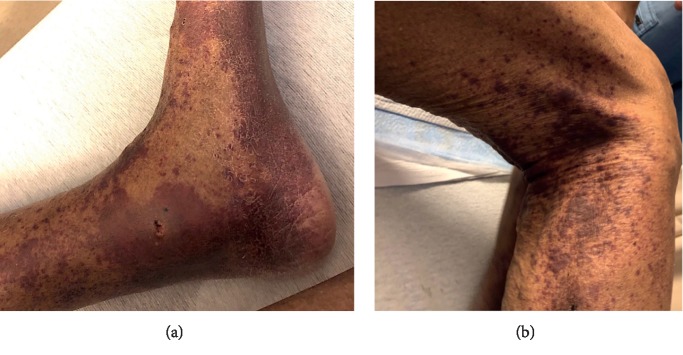
Purpuric rash of the left lower extremity (a) and the right popliteal region (b).

Apixaban is a rare cause of immune-complex small-vessel vasculitis. This is also referred to as leukocytoclastic vasculitis (LCV). LCV is a histopathologic diagnosis given to a small-vessel vasculitis where immune complexes deposit in the vessel walls. To our knowledge, there is only one other reported case due to apixaban in the literature [1]. We describe a case of leukocytoclastic vasculitis secondary to apixaban (Figure 1).

## 2. Methods

Case report and review of the literature.

## 3. Case

A 95-year-old male presented with a one-day history of a diffuse, mildly tender but nonpruritic palpable purpura of his feet, ankles, and lower legs (Figure 1). The patient reported that the rash rapidly spread up his legs, to his back, flanks, and buttocks. The lesions were well demarcated, ranged from dark red to violet in color and varied in size. His past medical history was significant for hypertension, hyperlipidemia, hypothyroidism, congestive heart failure, previous coronary artery bypass graft, and nonvalvular atrial fibrillation. His home medications included apixaban, aspirin, finasteride, furosemide, isosorbide mononitrate, mirabegron, thyroid (Armour thyroid) tablet, and tamsulosin. The patient had been transitioned from warfarin to apixaban twelve days prior due to the burden of associated monitoring on warfarin. He had no other medication changes. He denied mucosal bleeding, melena or hematochezia. He denied any preceding chemical exposures, insect bites, fevers, or arthralgias.

On admission, he was afebrile, hypertensive to 150/88 mmHg, but otherwise, vitals were within normal limits. In addition to his new rash, his physical exam was significant for 3/4 diastolic ejection murmur best heard at the left lower sternal border and mild bibasilar crackles on auscultation.

Apixaban was discontinued on admission as it was hypothesized to be a contributor to the vasculitis. His white cell count was 7.8k on admission with 0.9% eosinophils. He also had thrombocytopenia, with a platelet count of 63,000. Significant labs included erythrocyte sedimentation rate 33 mm/h, low complement (C3: 23, C4: <8), and negative cryoglobulins. His ANA was 1 : 160, with rheumatoid factor of 13. Screening test for hepatitis C antibody was negative. Serum and urine protein electrophoresis showed no evidence of paraprotein. Inpatient infectious and autoimmune laboratory tests were otherwise negative. Biopsy of the lesion showed extensive purpura with superficial perivascular neutrophilic infiltrate and leukocytoclasis. Gram stain and Grocott stains were negative for bacterial or fungal involvement. Immunofluorescence was not completed on this specimen.

Given the concern for an inflammatory process, oral prednisone 40 mg daily was initiated, with a slow taper over five weeks. The purpuric rash and platelet count rapidly improved. Inflammatory markers and complement were not rechecked.

The patient was not formally tested with an allergy test during admission, but LCV secondary to apixaban was felt to be the most likely diagnosis. At the time of discharge, he was placed back on warfarin. At his follow-up visit, he had no further evidence of vasculitis and was tolerating warfarin.

## 4. Discussion

To our knowledge, this is the second reported case of apixaban-related leukocytoclastic vasculitis (LCV) in the literature [1]. LCV is a small-vessel vasculitis, where immune complexes are deposited in small-vessel walls, particularly involving the dermal postcapillary venules. The associated immune response results in loss of vessel wall integrity and extravasation of erythrocytes, resulting in the characteristic purpura [2]. The cutaneous manifestations often present in the lower extremities and buttocks as was the case in this patient [3]. When small-vessel vasculitis is suspected, punch biopsy should be completed as early as possible (within 24–48 hours of symptom onset) to maximize the diagnostic yield. Direct immunofluorescence can evaluate for the presence of immunoglobulins (such as IgA) which can better characterize the vasculitis and should be done within 8–24 hours of the rash [4]. Direct immunofluorescence was not completed on this patient's biopsy specimen, despite the biopsy being completed within approximately 16 hours after the rash developed. The reason for this was not specified by the pathologist. Drug-induced LCV is a diagnosis of exclusion after infectious, autoimmune, and inflammatory conditions have been excluded [3]. Almost 30% of all cases of LCV are drug-induced [5]; however, anticoagulants are a rare cause of LCV [3]. Complement levels should be checked after resolution of the rash to determine if the initial rash was an early manifestation of a systemic syndrome such as systemic lupus erythematosus (SLE) [6].

The interval between exposure to the offending agent and onset of the purpuric rash is variable. On review of the literature, the majority occurred between 7 and 14 days after initiation of the causative medication; however, some reactions appeared after years of use [1–3, 7–9]. The management of other cases of anticoagulant-induced LCV involved discontinuing the causative drug, with or without the initiation of immunosuppressive therapies [1–3, 7–9]. There is a clear role for immunosuppressive therapies in cases involving skin necrosis, severe systemic vasculitis, or refractory cases [2]. Various case reports of anticoagulant-induced LCV suggest that patients can be safely initiated on an alternative anticoagulant due to the difference in molecular structures between these medications [1]. However, high-level evidence in this area is lacking [1].

Our case of apixaban-induced LCV highlights a rare complication of a common medication, which is important for physicians to be aware of.

## 5. Conclusion

Apixaban is a rare but important cause of leukocytoclastic vasculitis. In cases isolated to the skin, treatment is supportive and consists of withdrawal of the offending medication. Patients should be transitioned to a different anticoagulant. In more complicated cases involving skin necrosis or severe systemic vasculitis, there is a role for immunosuppressive therapy.
